# Proteome Analysis for Downstream Targets of Oncogenic KRAS - the Potential Participation of CLIC4 in Carcinogenesis in the Lung

**DOI:** 10.1371/journal.pone.0087193

**Published:** 2014-02-04

**Authors:** Koji Okudela, Akira Katayama, Tetsukan Woo, Hideaki Mitsui, Takehisa Suzuki, Yoko Tateishi, Shigeaki Umeda, Michihiko Tajiri, Munetaka Masuda, Noriyuki Nagahara, Hitoshi Kitamura, Kenichi Ohashi

**Affiliations:** 1 Department of Pathology, Yokohama City University Graduate School of Medicine, 3-9, Future, Kanazawa-ku, Yokohama, Japan; 2 Department of Biochemistry/Cell Biology, Nippon Medical University, 1-1-5, Sendagi, Bunkyo-ku, Tokyo, Japan; 3 Department of Surgey, Yokohama City University Graduate School of Medicine, 3-9, Future, Kanazawa-ku, Yokohama, Japan; 4 Division of General Thoracic Surgery, Kanagawa Prefectural Cardiovascular and Respiratory Center Hospital, 6-16-1, Tomioka-higashi, Kanazawa-ku, Yokohama, Japan; 5 Department of Enviromental Medicine, Nippon Medical University, 1-1-5, Sendagi, Bunkyo-ku, Tokyo, Japan; Cincinnati Children's Hospital Medical Center, United States of America

## Abstract

This study investigated the proteome modulated by oncogenic KRAS in immortalized airway epithelial cells. Chloride intracellular channel protein 4 (CLIC4), S100 proteins (S100A2 and S100A11), tropomyosin 2, cathepsin L1, integrinsα3, eukaryotic elongation factor 1, vimentin, and others were discriminated. We here focused on CLIC4 to investigate its potential involvement in carcinogenesis in the lung because previous studies suggested that some chloride channels and chloride channel regulators could function as tumor suppressors. CILC4 protein levels were reduced in some lung cancer cell lines. The restoration of CLIC4 in lung cancer cell lines in which CLIC4 expression was reduced attenuated their growth activity. The immunohistochemical expression of the CLIC4 protein was weaker in primary lung cancer cells than in non-tumorous airway epithelial cells and was occasionally undetectable in some tumors. CLIC4 protein levels were significantly lower in a subtype of mucinous ADC than in others, and were also significantly lower in KRAS-mutated ADC than in EGFR-mutated ADC. These results suggest that the alteration in CLIC4 could be involved in restrictedly the development of a specific fraction of lung adenocarcinomas. The potential benefit of the proteome modulated by oncogenic KRAS to lung cancer research has been demonstrated.

## Introduction

Lung cancer is one of the most common causes of cancer-related death in the developed world [1], [2]. If primary tumors are successfully removed surgically removed, the incidence of recurrence remains high [1], [2]. Although some lung tumors are sensitive to conventional chemotherapeutic agents or certain molecular targeting agents, many are not [3], [4]. Thus, further understanding of the molecular basis of carcinogenesis in the lung is needed in order to develop novel therapeutic strategies.

Our previous studies identified important molecules involved in carcinogenesis in the lung through a comprehensive search for the downstream targets of oncogenic *KRAS* [3], [5]. *KRAS* is known to transmit potential signals that cause opposing biological effects. Some downstream targets may be growth suppressors while others may be accelerators [3]. A disruption in the balance between these effects may occasionally result in a neoplastic transformation and also promote the progression of carcinogenesis.

Such downstream targets were previously shown to be involved in not only in the development of lung cancers with KRAS mutations, but also of those without KRAS mutations [3], [5]. These findings indicated that investigating the downstream targets of oncogenic KRAS reveal the common important molecular basis of lung cancer.

The present study examined the post-translational expression profile (proteome) of oncogenic KRAS-transduced airway epithelial cells and identified some downstream molecules. We focused on CLIC4, a member of the chloride intracellular channel protein family [6]–[8], because previous studies suggested that some chloride channels and chloride channel regulators could function as tumor suppressors [5]. To verify the potential involvement of CLIC4 in carcinogenesis in the lung, we here examined lung cancer cell lines and primary human lung cancers for the expression of CLIC4, and analyzed the correlation between its expression levels and different clinicopathologic parameters.

## Materials and Methods

### Cell lines and culture

An immortalized human airway epithelial cell line (16HBE14o, Simian virus 40 (SV40)-transformed human bronchial epithelial cells) described by Cozens AL et al. (1994) [9] was kindly provided by Grunert DC (California Pacific Medical Center Research institute). A sub-clone of 16HBE14o cells, described as NHBE-T in this study, was used in the present study. Human lung cancer cell lines (A549, H358, H2087, H1819, H441, and H1299) and a human embryonic kidney cell line (HEK293T) were purchased from the American Type Culture Collection (ATCC, Manassas, VA). The human lung cancer cell lines, Lu135 and Lu139 were purchased from Riken Cell Bank (Tsukuba, Japan). PC9 and HARA were purchased from Immunobiological Laboratories Co. (Gunma, Japan). TKB5, TKB6, TKB7, TKB8, TKB8, TKB14, and TKB20 were established and gifted by Dr. H Kamma via Dr. T Yazawa (Kyorin University School of Medicine, Tokyo, Japan) [10]. The Ethics Committee of Yokohama City University approved the experimental protocol using these cell lines.

### Plasmid construction

The construction of pro-retrovirus vectors bearing wild-type (pQCXIH/KRAS G12) and mutated KRAS (pQCXIH/KRAS V12) has been described elsewhere [11]. CLIC4 cDNA (NM_013943) was PCR-amplified and inserted into the pQCXIP (BD Clontech, Palo Alto, CA) pro-retrovirus vector. Vectors bearing a sense and antisense strand of cDNA were obtained. The pro-retrovirus vector pSINsi bearing a short hairpin RNA for the knockdown of CLIC4 was ordered from Takara Bio Inc.

### Retroviral-mediated gene transfer

pQCXIH/P-based expression plasmid vectors and the pCL10A1 retrovirus-packaging plasmid vector (IMGENEX, San Diego, CA) were cotransfected into HEK293T cells with Lipofectoamine 2000 reagent (Invitrogen, Carlsbad, CA), and conditioned medium was then recovered as a retroviral vector solution. The desired genes were transduced by incubating cells with the viral solution containing 10 µg/ml of polybrene (Sigma, St. Louis, MO). Cells stably expressing the desired genes were selected with 300 µg/ml of Hygromycin D or 5.0 µg/ml of Puromycin (Invitrogen) for 3 days. Pooled clones were used for biological analyses as follows.

### Proteome analysis

Whole cell protein was labeled with Cy2, Cy3, or Cy5 using a protein-labeling kit (Amersham, Piscataway, NJ). Protein lysates were prepared from mock-transduced NHBE-T cells, KRAS G12-transduced NHBE-T cells, and KRAS V12-transduced NHBE-T cells. Two-dimensional electrophoresis was performed using a horizontal electrophoresis system (Maltiphor II) (Amersham) according to the manufacturer's instructions. Briefly, an equal amount of a protein sample was subjected to first-dimensional isoelectric focusing, followed by second dimensional sodium dodecyl sulfate-polyacrylamide gel electrophoresis. Proteins on differentially expressed spots were identified by LC-MS/MS analysis. The details of this method have been described elsewhere [12], [13]

### Western blotting

Whole cell lysates were subjected to SDS-polyacrylamide gel electrophoresis, and transferred onto PVDF membranes (Amersham). The membranes were then incubated with nonfat dry milk in Tris-buffered saline containing Tween-20 (TBS-T) to block non-immunospecific protein binding, and then with a primary antibody against KRAS (Santa Cruz, Santa Cruz, CA), CLIC4 (Santa Cruz), Smad2/3 (Cell Signaling Technology, Danvers, MA), phosphorylated Smad2/3 (Cell Signaling Technology), caspase-3 (Cell Signaling Technology), or β-actin (Sigma). After washing with TBS-T, the membranes were incubated with animal-matched HRP-conjugated secondary antibodies (Amersham). Immunoreactivity was visualized with an enhanced chemiluminescence system (Amersham).

### Primary Lung Cancer

All 235 tumors examined (180 adenocarcinoma (ADC), 39 squamous cell carcinomas (SQC), and 16 large cell carcinomas (LCC)) were removed by radical surgical resection at the Kanagawa Cardiovascular and Respiratory Center (Yokohama, Japan). The research plan was approved by the Ethics Committees of Yokohama City University and Kanagawa Prefectural Cardiovascular and Respiratory Center Hospital. Written informed consent for research use was obtained from all subjects providing materials. Histological types and disease stages were determined according to the International TNM Classification System (seventh edition of the UICC) [14].

### Immunohistochemistry

Tumor sections were cut from formalin-fixed, paraffin-embedded tissue blocks. Sections were deparaffinized, rehydrated, and incubated with 3% hydrogen peroxide, followed by 5% goat serum to block endogenous peroxidase activities and non-immunospecific protein binding. Sections were boiled in citrate buffer (0.01 M, pH 6.0) for 15 minutes to retrieve masked epitopes and then incubated with a primary antibody against CLIC4 (Santa Cruz) or Ki-67 (DAKO, Ely, UK). Immunoreactivity was visualized using an Envision detection system (DAKO), and nuclei were counterstained with hematoxylin. CLIC4 immunohistochemical expression levels were evaluated by a scoring system as described in the Results section. The labeling index of Ki-67 was calculated as the proportion of positive nuclei cells by counting 500–1000 cancer cells. The Ki-67 labeling indices of <10% and ≥10% were classified as low and high levels, according to the results of our previous study [4].

### Colony formation assays

Cells (2.5×10^4^) were seeded onto a 10-cm culture dish (Iwaki, Tokyo, Japan), and grown for 10 days. Cells were fixed with methanol and Giemsa-stained, and colonies visible in scanned photographs were counted.

### Growth curve assays

Cells (2.5×10^5^) were seeded onto a 10-cm culture dish, and grown to a semi-confluent state for the appropriate number of days. Cells were counted and 2.5×10^5^ cells were reseeded onto a 10-cm dish. Several passages were repeated in the same manner. The sum of population doublings at each point was calculated by the formula ΣPDL_n_ = log_2_ (count_n_/2.5×10^5^)+ΣPDL_n-1_.

### Soft agar colony formation assay

Cells (1.25×10^4^) were grown in 1 ml of DMEM-based 0.3% agar (Agar Noble; Becton Dickinson, Sparks, MD) containing 10% FBS in a 3.5-cm culture dish (Iwaki) for 4 weeks. The agar was fixed with a buffered 4% paraformaldehyde solution, and colonies visible in scanned photographs were counted.

### Statistical Analysis

Differences in the mean values of the immunohistochemical score for CLIC4 among the groups classified based on various clinicopathologic subjects were analyzed by one-way ANOVAs. Recurrence curves were plotted using the Kaplan-Meier method, and the absolute risk of recurrence at five years was estimated from these curves. Differences in the disease-free survival (DFS) span and rate were analyzed using the log-rank test. P values less than 0.05 were considered significant. Statistical analyses were performed using SPSS software (SPSS for Windows Version 10.0; SPSS; Chicago, IL, USA).

## Results

### Protein expression profiling modulated by oncogenic KRAS

**Figure 1:**
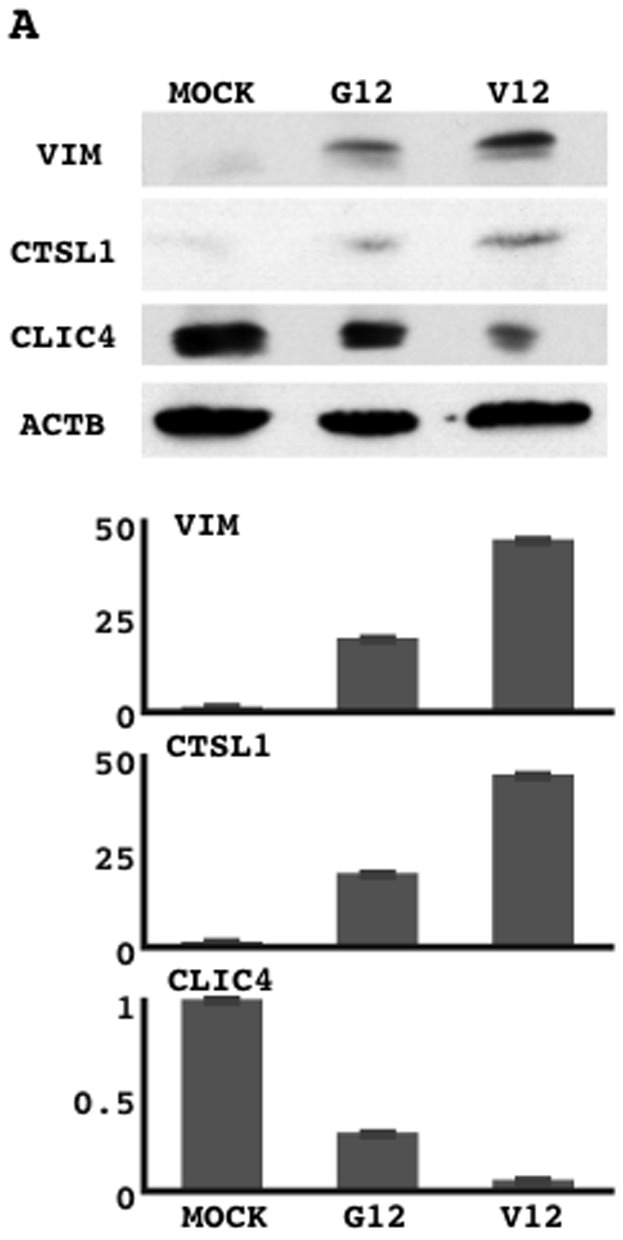
Protein lysates from mock-transduced NHBE-T cells (MOCK), wild-type KRAS G12-transduced NHBE-T cells (KRAS/G12), and mutant KRAS V12-transduced cells (KRAS/V12), were labeled with the Cy2 (violet), Cy3 (green), and Cy5 (orange red) fluorescent dyes, respectively. Equal amounts of labeled protein from MOCK and KRAS/V12 (left panel) or from KRAS/G12 and KRAS/V12 (right panel) were mixed and subjected to 2-dimensional electrophoresis (A). The signal intensities of all the spots detected were comprehensively evaluated and subjected to hierarchical clustering analysis (Ward's method) (B). Whole cell protein lysates were subjected to Western blot analysis for the expression of KRAS and β-actin (ACTB) (C).

**Figure 2:**
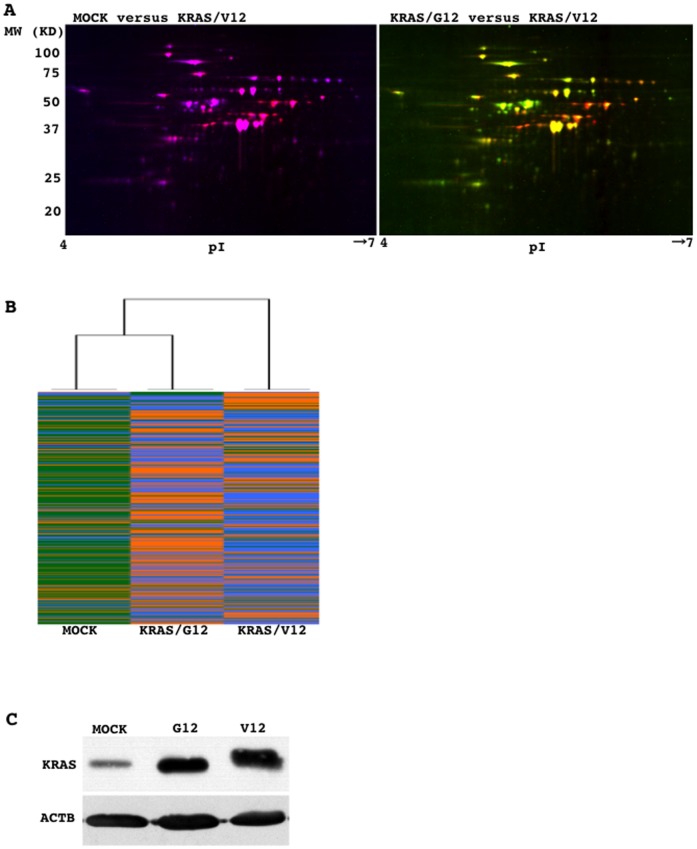
Protein lysates from mock-transduced NHBE-T cells (MOCK), wild-type KRAS G12-transduced NHBE-T cells (KRAS/G12), and mutant KRAS V12-transduced cells (KRAS/V12) were subjected to Western blot analysis for vimentin (VIM), cathepsin L1 (CASTL1), CLIC4, and β-actin (ACTB) (A). The signal intensities of the bands were evaluated by NIH imaging. VIM, CSTL1, and CLIC4 levels were normalized to those of ACTB. Normalized levels are shown (B).

Two-dimensional electrophoresis (representative results are shown in Fig. 1A) discriminated 1662 spots. A comprehensive evaluation of the signal intensities of these spots revealed that oncogenic KRAS-transduced cells had different expression profiles to those of empty vector- and wild-type KRAS-transduced cells, as KRAS/V12-transduced cells were classified into a distant branch on a dendrogram described by hierarchical clustering analysis of the Ward's method (Fig. 1B). Proteins that were more than 2-fold higher or lower in oncogenic KRAS-transduced cells than in both mock- and wild-type KRAS-transduced cells are listed (Table 1). The successful expression of the transduced KRAS gene was confirmed by Western blot analysis (Fig. 1C). Among the molecules listed, a focus was placed on VIM, CTSL1, and CLIC4, because they were repeatedly identified (Table 1). This finding was confirmed by Western blot analysis (Fig. 2A). As mentioned in the Introduction section, previous studies have suggested that some chloride channels and chloride channel regulators may function as tumor suppressors [5], [15]–[18]. Thus, the present study focused on CLIC4, a member of the intracellular chloride channel family [6]–[8], to further investigate its potential involvement in carcinogenesis in the lung.

**Table 1:** Differentially expressed or modified proteins by oncogenic KRAS.

				Signal Intensity			Signal Ratio	
Protein	Accession	Map	P value	MCK	G12	V12	V12/G12	V12/G12
Increasing spot density								
S100A2	NM_005978	1q21.33	0.00012140	0.400	1.005	1.665	1.657	4.163
VIME	NM_003380	10p13	0.00000091	0.368	0.754	1.424	1.889	3.870
EEF2	NM_001961	19p13.3	0.00000313	0.454	0.922	1.515	1.643	3.337
GC	NM_000583	4q12-q13	0.00100000	0.480	0.934	1.582	1.694	3.296
VIME	NM_003380	10p13	0.00097490	0.473	0.758	1.378	1.818	2.913
VIME	NM_003380	10p13	0.00000583	0.545	0.854	1.494	1.749	2.741
CTSL1	NM_001912	9q21.33	0.00034140	0.635	0.708	1.708	2.412	2.690
VCP	NM_007126	9p13.3	0.00200000	0.599	1.010	1.551	1.536	2.589
VIME	NM_003380	10p13	0.00027980	0.508	0.807	1.292	1.601	2.543
ENOA	NM_001428	1p36.2	0.00013390	0.641	0.936	1.560	1.667	2.434
S100A11	NM_005620	1q21	0.00200000	0.569	0.761	1.291	1.696	2.269
VIME	NM_003380	10p13	0.00013750	0.655	0.864	1.439	1.666	2.197
VIME	NM_003380	10p13	0.00012950	0.604	0.884	1.324	1.498	2.192
VIME	NM_003380	10p13	0.00015590	0.688	0.891	1.492	1.675	2.169
CTSL1	NM_001912	9q21.33	0.00100000	0.730	0.802	1.551	1.934	2.125
VIME	NM_003380	10p13	0.00007393	0.608	0.810	1.277	1.577	2.100
Decreasing spot density								
CLIC4	NM_013943	1p36.11	0.00001027	1.103	0.882	0.513	0.582	0.465
TPM2	NM_003289	9p13	0.00003761	1.070	0.742	0.482	0.650	0.450
CLIC4	NM_013943	1p36.11	0.00010130	1.364	1.043	0.591	0.567	0.433
ITGA3	NM_002204	17q21.33	0.00082800	1.230	1.110	0.519	0.468	0.422
ATP5B	NM_001686	12q13.13	0.00004170	1.417	0.938	0.587	0.626	0.414

Accession, gene bank accession number; Map, chromosome locus;

MCK, mock-transduced NHBE-T; G12, wild-type KRAS-transduced NHEB-T;

V12, oncogenic mutant KRAS/V12-transduced NHBE-T.

### CLIC4 protein expression in lung cancer cell lines

**Figure 3:**
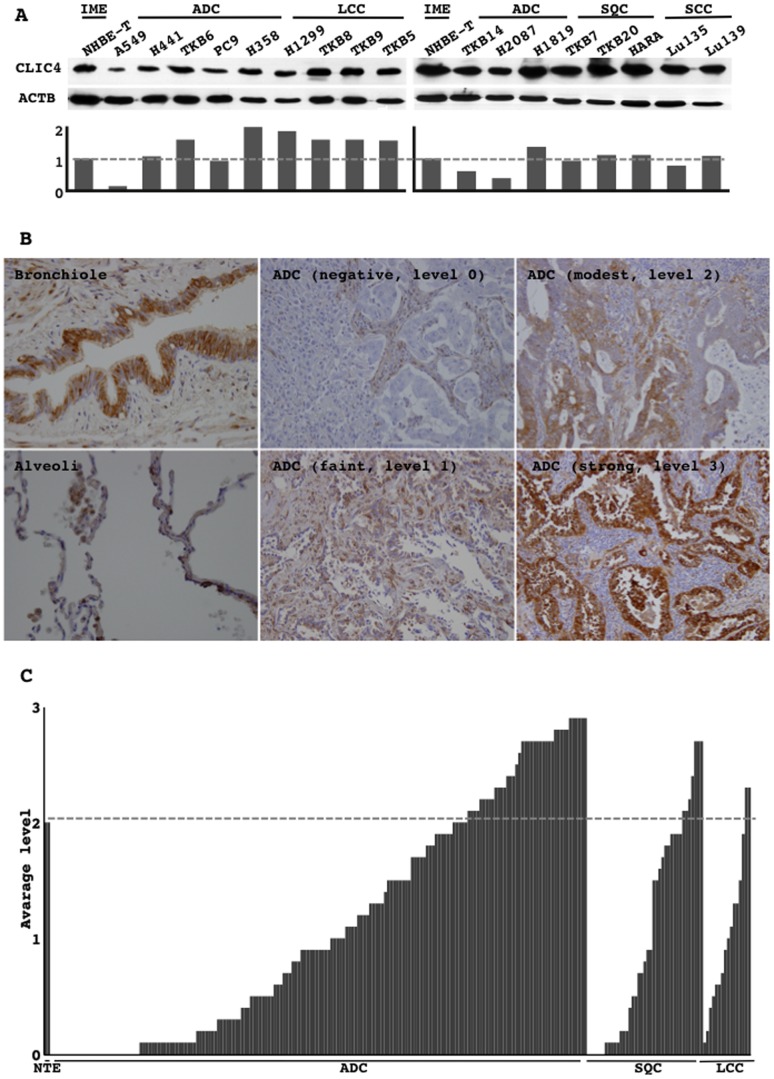
Protein lysates from lung cancer cell lines were subjected to Western blot analysis for CLIC4 and β-actin (ACTB) (A upper panel). The signal intensities of the bands were evaluated by NIH imaging. CLIC4 levels were normalized to those of ACTB. Normalized levels are shown (A lower panel). The expression of the CLIC4 protein in tumors and non-tumorous epithelia from lung cancer patients undergoing surgical resection were immunohistochemically examined. Representative photographs from the bronchioles, alveoli, and tumors in which CLIC4 expression was negative, faint, modest, and strong are shown (B). CLIC4 expression levels in all the tumors examined are shown (C). The dashed line indicates the level of non-neoplastic epithelia cells (NTE). ADC, adenocarcinoma; SQC, squamous cell carcinoma; LCC, large cell carcinoma.

Among the lung cancer cell lines examined, some cell lines (A549, TKB14, and H2087) had lower CLIC4 protein levels than those in immortalized non-cancerous airway cells (Fig. 3A).

### CLIC4 protein expression in primary lung cancers

The CLIC4 protein was expressed in the cytoplasm and plasma membrane of airway epithelial cells in the bronchioles and alveoli (Fig. 3B). The level of CLIC4 protein expression was varied in neoplastic cells even in individual tumors. Some expressed CLIC4 at an equivalent level or more strongly than that of the bronchioles and alveoli (Fig. 3B), while expressed CLIC4 faintly or at an undetectable level (Fig. 3B). Expression levels were classified into negative (level 0), faint (level 1), modest (level 2), and strong (level 3). The faint level was defined as weaker than the level in the bronchioles and alveoli, but not negative. The modest level was defined as a level equivalent to that in the bronchioles and alveoli. The strong level was defined as an unequivocally stronger level than that in the bronchioles and alveoli. The immunohistochemical expression score was determined as an average level in the maximal tumor section (if 30%, 10%, 50%, and 10% of neoplastic cells in the maximal section of tumor were negative, faint, modest, and strong levels, respectively, the average level was calculated as “1.4 = 0.3×0+0.1×1+0.5×2+0.1×1”). The average levels in all the tumors examined are shown (Fig. 3C). Neoplastic cells generally appeared to express CLIC4 at a lower level than that in bronchial and alveolar epithelial cells (Fig. 3C). No significant difference in CLIC4 levels was observed among histological subtypes. However, CLIC4 levels in ADCs were lower in the mucinous subtype and also decreased with the progression of the histological grade (Table 2). CLIC4 levels were higher in EGFR-mutated ADCs than in KRAS-mutated ADCs (Table 2).

**Table 2:** Correlation between CLIC4 expression and pathologic subjects.

	Expression score
*Histology (235)	
ADC (180)	1.098±0.981
*Grade	
WEL (115)	1.208±0.975
MOD (43)	0.856±0.942
POR (22)	0.995±1.065
*Subtype	
BAC (89)	1.246±0.947
ACI (37)	0.935±0.935
PAP (14)	1.333±1.049
SOL (22)	1.023±1.107
MUC (14)	0.400±0.708
SQC (39)	1.049±0.937
Grade	
WEL (2)	2.200±0.707
MOD (34)	1.038±0.930
POR (3)	0.400±0.436
LCC (16)	1.044±0.687
Vascular involvement/ADC (180)	
Present (16)	1.312±1.174
Absent (164)	1.077±0.962
Lymphatic canal involvement/ADC (180)	
Present (17)	0.935±0.952
Absent (163)	1.115±0.985
Ki-67 labeling index/ADC (180)	
Low level (<10%) (95)	0.979±0.980
High level (≥10%) (85)	1.119±0.983
*Oncogenic mutation/ADC (155)	
KRAS (15)	0.973±1.003
EGFR (46)	1.650±0.881
NONE (94)	0.931±0.103

MUC, mucinous adenocarcinoma; NONE, cases without KRAS or EGFR mutations.

* Significant in a one-way ANOVA analysis; grade (WEL versus MOD, P = 0.0437); subtype (MUC versus BAC, P<0.0018; MUC versus PAP, P = 0.0077); oncogenic mutation (EGFR versus KRAS, P = 0.0154; EGFR versus NONE, P<0.0001); ADC, adenocarcinoma; SQC, squamous cell carcinoma; LCC, large cell carcinoma; WEL, well differentiated; MOD, moderately differentiated; POR, poorly differentiated carcinomas; BAC, bronchioloalveolar carcinoma; ACI, acinar adenocarcinoma; PAP, papillary adenocarcinoma; SOL, solid adenocarcinoma;

### Effect of CLIC4 on cell growth activity

**Figure 4:**
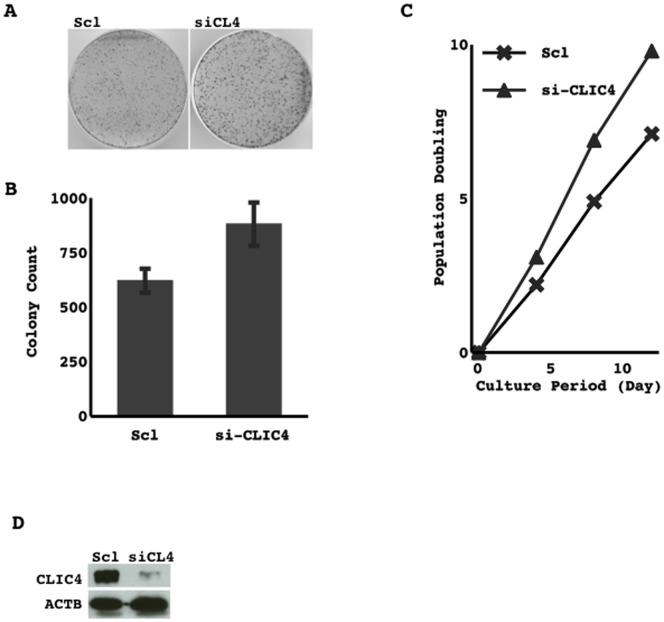
Transduction of scrambled RNA (scl) and siRNA for CLIC4 (siCL4) were performed. Following brief selection for 3 days, surviving cells were harvested and counted, and 2.0×10^4^ cells were reseeded onto a 10-cm dish. Cells were methanol-fixed and Giemsa-stained after 14 days culture (A). The means and standard deviations (error bars) of the colony counts from triplicate experiments are presented (B). The cells selected were grown and passed several times. Cumulated population doublings are presented (C). Cells harvested immediately after the selection process were examined for the expression of CLIC4 by Western blotting (D).

The knockdown of CLIC4 in an immortalized non-cancerous cell line (NHBE-T) elevated growth activity because it increased clonogenicity and shortened the doubling time (Fig. 4).

**Figure 5:**
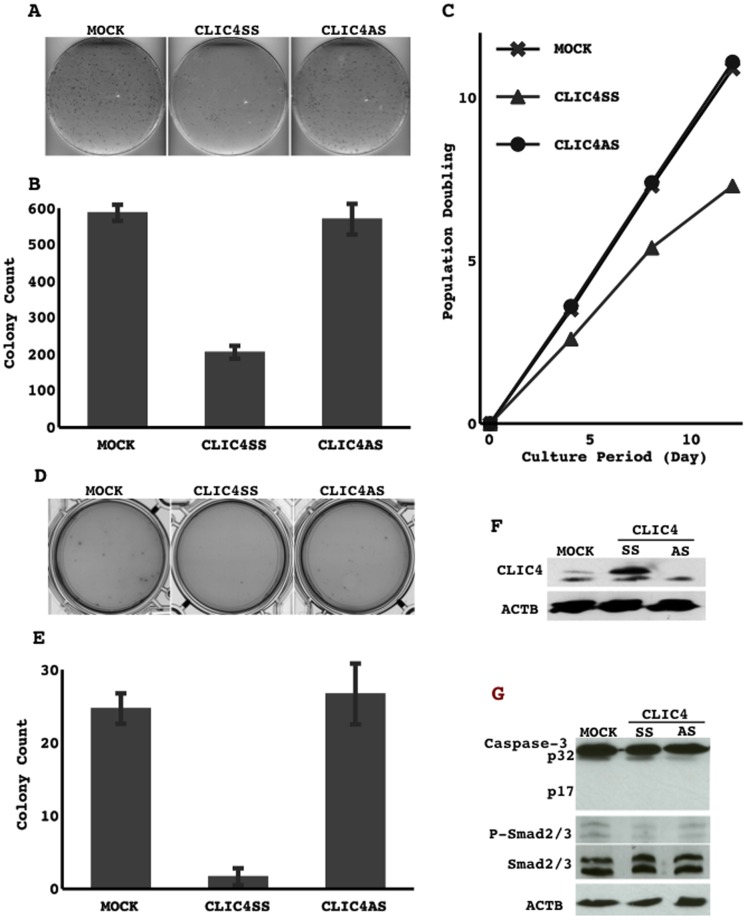
Transduction of empty vector (MOCK), sense strand of CLIC4 (CLIC4SS), and antisense strand of miR-31 (CLIC4AS) were performed. Following a brief selection for 3 days, surviving cells were harvested and counted, and 2.0×10^4^ cells were reseeded onto a 10-cm dish. Cells were methanol-fixed and Giemsa-stained after 14 days culture (A). The means and standard deviations (error bars) of colony counts from triplicate experiments are presented (B). Cells selected were grown and passed several times. Cumulated population doublings are presented (C). The selected cells (1.25×10^4^) were cultured and grown in 1 ml of DMEM-based 0.3% agar containing 10% FBS in 3.5-cm culture dishes for 4 weeks. The agars were fixed with a buffered 4% paraformaldehyde solution (D). The means and standard deviations (error bars) of colony counts from triplicate experiments are presented (E). Cells harvested immediately after the selection process were examined for the expression of CLIC4, caspase-3, phosphorylated Smad2/3, Smad 2/3, and β-actin (ACTB), by Western blotting (F and G).

The forced expression of CLIC4 reduced clonogenicity, prolonged the doubling time, and attenuated anchorage growth activity in some NSCLC cell lines in which the expression of CILC4 was weaker (A549 and H2087). Representative results from A549 cells are shown (Fig. 5A–E). The exogenous expression of CLIC4 was verified by Western blotting (Fig. 5F).

CLIC4 has been suggested to prolong the activation of Smad2/3 to induce apoptosis by inhibiting its dephosphorylation through a selective phosphatase (PPM1a) [19], [20]. Thus, the potential involvement of this mechanism in the CLIC4-induced suppression of growth was investigated. However, the cleavage of caspase-3 and level of phosphorylated Smad2/3 was not modulated by the forced expression of CLIC4 (Fig. 5G), which indicates that some other mechanisms may be controlling cell growth through CLIC4.

### Association between CLIC4 expression and disease-free-survival

**Figure 6:**
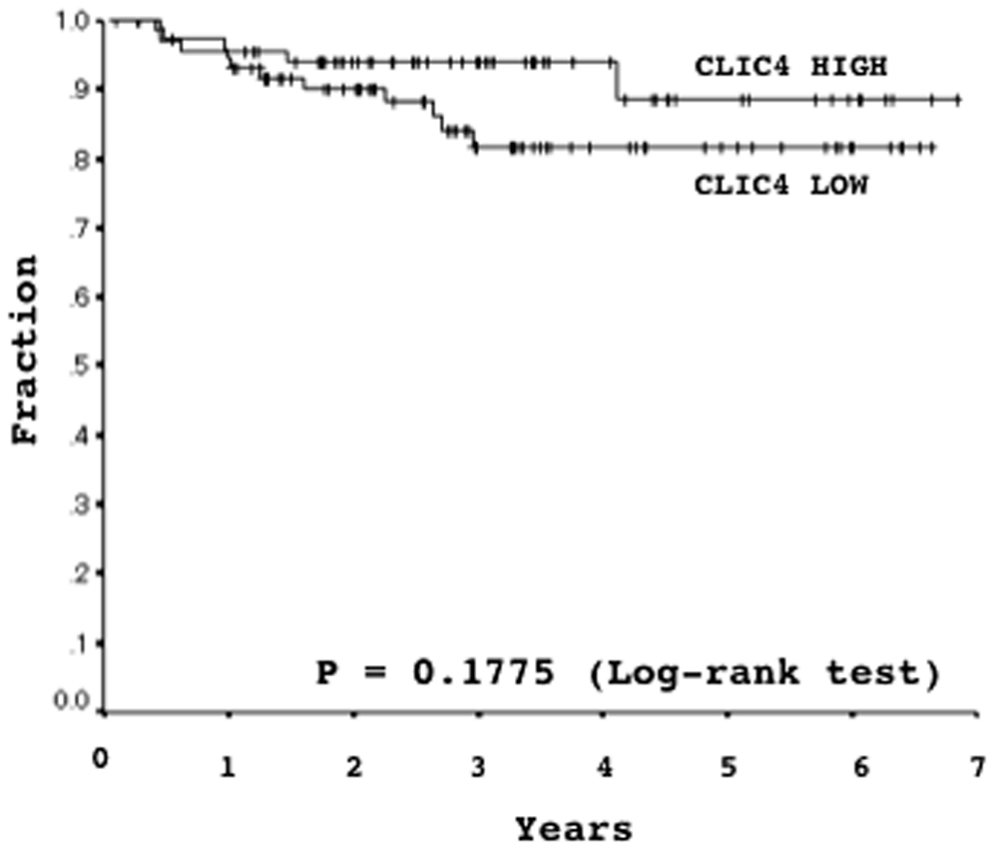
The relationship between CLIC4 levels and disease-free survival in stage I lung adenocarcinomas was analyzed by the Log-rank test (P = 0.1775). Kaplan-Meier survival curves are shown (five-year disease-free survival rates were 81.70% and 88.71% in CLIC4 low- and high-expressers, respectively.

A total of 144 patients were available for disease-free survival analysis. The averaged level of <9.5% and ≥9.5% were classified as low and high, based on a receiver operating characteristic curve (area under the curve 0.615, 95% confidential interval 0.460–0.769). Seventy-five patients (52.1%) were low expressers, and 69 (47.9%) were high expressers. The incidence of recurrence was slightly higher in low expresser than in the high expressers, while no significant differences were observed in disease-free survival was not significant between the high and low expressers (five-year disease-free survival rate, 88.71% in the high expressers versus 81.70% in the low expressers, P = 0.1775)(Fig. 6).

## Discussion

The present study investigated the proteome modulated by oncogenic KRAS in immortalized airway epithelial cells and identified potential downstream targets (Table 2). Chloride intracellular channel proteins (CLIC), S100 proteins, tropomyosins (TPM), cathepsins, α-integrins (ITGA), eukaryotic elongation factors (EEF), and vimentin (VIM) were also discriminated through analyses on the RAS-modulated proteome of different types of cells investigated in previous studies [21]–[31]. The results obtained suggested that these could be important molecules participating in RAS-mediated carcinogenesis. We here focused on CLIC4, a chloride intracellular channel protein [6]–[8], because previous studies suggested that some chloride channels and chloride channel regulators could function as tumor suppressors [5].

CILC4 protein levels were reduced in some lung cancer cell lines (Fig. 3A). The knockdown of CLIC4 in an immortalized non-cancerous cell line elevated growth activity (Fig. 4), and the restoration of CLIC4 in lung cancer cell lines in which CLIC4 expression was reduced attenuated their growth activity (Fig. 5), suggesting that CILC4 could be a tumor suppressor. Consistent with these results, the immunohistochemical expression of the CLIC4 protein was also weaker in primary lung cancer cells than in tumorous airway epithelial cells, and was occasionally undetectable in some tumors (Fig. 3B). CLIC4 levels decreased with the progression of the histological grade among ADCs. The incidence of recurrence was slightly higher in low expressers than in high expressers. However, no significant differences were observed in disease-free survival (Fig. 6). These results indicate that CLIC4 may function as a tumor suppressor in the progression process of carcinogenesis of lung ADC. Moreover, CLIC4 levels were significantly lower in a subtype of mucinous ADC than in others, and were also significantly lower in KRAS-mutated ADCs than in EGFR-mutated ADCs (Table 2). Mucinous ADC is known to be a subtype that is preferentially affected by KRAS mutations [32]–[34]. These results appear to be reasonable and suggest that an alteration in CLIC4 could be involved in restrictedly development of a specific fraction of ADCs. The different subtypes of ADCs may develop through the accumulation of subtype-specific molecular alterations.

CLIC4 belongs to the family of chloride intracellular channel proteins, which is composed of seven family members, p64, CLIC1 to CLIC5, and parchorin [19], [35], [36]. CLIC4 is multifunctional and is known to suppress cell growth through the transforming growth factor β (TGFβ) signaling pathway and promote cellular differentiation through the protein kinase δ (PKCδ) signaling pathway [7], [37], [38]. Moreover, CLIC4 is a direct transcriptional target for p53 and c-Myc, and is essential for the induction of apoptosis [35]. Thus, CLIC4 has been suggested to have a suppressive effect on tumor development. Previous studies demonstrated that CLIC4 protein levels were reduced in neoplastic cells in several types of human malignancies including lung cancer [39]. This finding is compatible with the results of the present study, and supports our hypothesis that CLIC4 may be a tumor suppressor in carcinogenesis in the lung.

In summary, this study revealed the proteome modulated by oncogenic KRAS, and focused on CLIC4 to investigate its potential involvement in carcinogenesis in the lung. These results suggest that CLIC4 could be a tumor suppressor, and its downregulation may promote carcinogenesis in a specific fraction of lung cancers. The potential benefit of the proteome modulated by oncogenic KRAS to lung cancer research has been demonstrated.
